# A Generalized Regression Neural Network Model for Predicting the Curing Characteristics of Carbon Black-Filled Rubber Blends

**DOI:** 10.3390/polym14040653

**Published:** 2022-02-09

**Authors:** Ivan Kopal, Ivan Labaj, Juliána Vršková, Marta Harničárová, Jan Valíček, Darina Ondrušová, Jan Krmela, Zuzana Palková

**Affiliations:** 1 Department of Numerical Methods and Computational Modeling, Faculty of Industrial Technologies in Púchov, Alexander Dubček University of Trenčín, Ivana Krasku 491/30, 020 01 Púchov, Slovakia; ivan.kopal@tnuni.sk (I.K.); jan.krmela@tnuni.sk (J.K.); 2 Department of Materials Technologies and Environment, Faculty of Industrial Technologies in Púchov, Alexander Dubček University of Trenčín, Ivana Krasku 491/30, 020 01 Púchov, Slovakia; ivan.labaj@tnuni.sk (I.L.); darina.ondrusova@tnuni.sk (D.O.); 3 Department of Material Engineering, Faculty of Industrial Technologies in Púchov, Alexander Dubček University of Trenčín, Ivana Krasku 491/30, 020 01 Púchov, Slovakia; juliana.vrskova@tnuni.sk; 4 Automation, Informatics and Physics, Institute of Electrical Engineering, Faculty of Engineering, Slovak University of Agriculture in Nitra, Tr. A. Hlinku 2, 949 76 Nitra, Slovakia; jan.valicek@uniag.sk (J.V.); zuzana.palkova@uniag.sk (Z.P.); 5 Department of Mechanical Engineering, Faculty of Technology, Institute of Technology and Business in České Budějovice, Okružní 10, 370 01 České Budějovice, Czech Republic

**Keywords:** rubber blends, curing process, modelling, generalized regression neural network

## Abstract

In this study, a new generalized regression neural network model for predicting the curing characteristics of rubber blends with different contents of carbon black filler cured at various temperatures is proposed for the first time The carbon black contents in the rubber blend and cure temperature were used as input parameters, while the minimum and maximum elastic torque, scorch time, and optimal cure time, obtained from the analysis of 11 rheological cure curves registered at 10 various temperatures, were considered as output parameters of the model. A special pre-processing procedure of the experimental input and target data and the training algorithm is described. Less than 55% of the experimental data were used to significantly reduce the total number of input and target data points needed for training the model. Satisfactory agreement between the predicted and experimental data, with a maximum error in the prediction not exceeding 5%, was found. It is concluded that the generalized regression neural network is a powerful tool for intelligently modelling the curing process of rubber blends even in the case of a small dataset, and it can find a wide range of practical applications in the rubber industry.

## 1. Introduction

Rubbers are among the most remarkable materials and are used in an extremely wide range of applications. However, to obtain their unique material properties—such as elasticity, high damping, impact resistance, hardness, long-term stability, and many others—the rubber compound or blend, which is generally a mixture of rubber, vulcanizing agent, accelerator, fillers and several additional ingredients, needs to be vulcanized (cured or cross-linked) to form an insoluble, cohesive, solid and shape-persistent rubber-based material. In the majority of all cases, rubbers are used in their vulcanized state, as vulcanizates or vulcanized rubbers above the glass transition temperature [1].

Traditionally, during vulcanization, the rubber blend is heated up to a temperature at which chemical bonds (cross-links) are formed in the chemical reactions between the long polymer chains of rubber and the vulcanizing agent, usually elemental sulphur, resulting in the formation of a spatial, 3D molecular network within the polymer matrix with different types of junctions [2]. In addition to sulphur, which is the most commonly used cross-linking agent for rubbers or elastomers in general, there are other vulcanizing systems, such as peroxides [3], ultraviolet light [4], electron beam [5,6], microwaves [7], resins [8], etc., which have become more important, in particular with the progressive development of synthetic rubbers [9].

In the rubber industry, it is essential to know the method of rubber cross-linking, in order to define its processability, curing time, and the final properties of the vulcanizate, which can be ensured by measuring its curing characteristics [1].

### 1.1. Curing Characteristics

Currently, the most efficient way to identify the curing characteristics of rubber blends (RBs) is to analyze the curing process using oscillating rheometers, which measure the changes in stiffness of an RB sample over time during its periodic shear loading at a constant frequency and cure temperature. The results are provided in the form of isothermal cure curves of elastic torque versus time. From these cure curves, the curing characteristics, such as minimum *M*_L_ and maximum *M*_H_ torque, scorch time *t*_s01_ and optimal cure time *t*_c90_, can be determined directly, and several derivative characteristics, such as curing speed ratio or cure rate coefficient, degree of cross-linking, thermo-plasticity, and others, can be computed [10].

It is well-known that RB curing is a highly sophisticated process that involves many influencing parameters. In terms of economic efficiency and processability, in addition to the vulcanizing system, the most important of them are the cure temperature, filler type and filler contents in the rubber matrix [1]. Nowadays, the curing process of RBs is most often carried out at temperatures from 140 to 210 °C, with sulphur as a vulcanizing agent and different types of carbon black (CB) in the role of a filler with contents ranging from 20 up to 400 phr in the blend [11].

In the context of the greening of the production, silica or its combinations with organic polysaccharides, such as cellulose and its various modifications, are increasingly used as fillers [12]. In most cases, organic fillers cannot be used as the majority filler in a rubber compound, mainly due to the loss of the utility properties of the vulcanizate. However, by the partial replacement of inorganic fillers with organic ones, the original properties of the compound can be substantially improved, such as by partial replacement of the commonly used inorganic CB filler by organic chitosan, which leads to an increase in the thermo-oxidative stability of the vulcanizate [13].

The cure temperature plays a crucial role in setting the technological parameters of the production. In fact, by its targeted control, it is possible to influence not only the energy intensity but also the ecological burden of the production process with minimal changes in the values of the main curing characteristics of the processed RBs, and thus also the desired utility properties of the vulcanizate [14]. The primary role of the filler is to cheapen RB and improve its processability. Currently, due to the most favorable price–performance ratio as well as the ability to directly influence the resulting values of curing characteristics through the so-called overheating effect of the blend, this role is usually played by the CB filler [15]. For these reasons, the influence of filler content and cure temperature on the progress and results of the curing process has long been a subject of widespread interest for many researchers and materials engineers. It is for these reasons that both of these factors of the curing process are selected as input parameters for the predictive model of curing characteristics of RBs, which is the main focus of the presented work.

### 1.2. Curing Process Modelling

Due to the large number of diverse parameters entering the curing process of RBs, its complex modelling by classical analytical tools, which would ultimately provide an accurate description of all parts of cure curves, is highly complicated. Some ad hoc models are well-described in the literature, e.g., in a doctoral thesis [16]. Most of these models are focused on the different sides of RB curing, such as the evolution of material parameters, the degree of curing, volume shrinkage, chemical composition, etc. However, in effect, a complex analytical model that includes all the features of RB curing does not exist at present.

Progress in artificial intelligence and machine learning over the last few decades [17] has shown that artificial neural networks (ANNs) are highly effective tools for the intelligent modelling of complex processes, which undoubtedly also include curing RBs. Their great advantage is that they are able to map any complex nonlinear relationship between process variables without prior information about the process or system models [18]. However, we found only three research papers published in prestigious journals dealing with this issue. For instance, in [19], the ability of three selected ANN architectures to predict the optimal cure time of 11 RBs at different cure temperatures in a model tire was studied. In the follow-up study [20] from the same authors, three different types of ANN and an adaptive neuro-fuzzy inference system (ANFIS) with an ANN architecture and the ANN learning technique were successfully used to predict the optimal cure time of the other 10 RBs at different cure temperatures. Another paper [21] developed a fast and accurate ANN model for rheological cure curve prediction of NR/SBR blends at various cure temperatures. In all three works, the whole cure curves were modelled using ANNs. Unlike these studies, the present study is, for the first time, devoted to ANN prediction not only of the optimal cure time, but of all of the four abovementioned curing characteristics of RBs with different contents of carbon black (CB) filler, cured at different temperatures, which is important for the rubber industry. At the same time, the whole cure curves were not used in creating the ANN model; instead, only their critical points (i.e., curing characteristics) determining the results of the curing process were employed. Such a novelty approach significantly increases the practical benefits of the model by reducing the number of necessary experimental measurements and increasing the efficiency of its creation and the training process [22].

### 1.3. Artificial Neural Networks

Artificial neural networks are intelligent, biologically inspired information processing systems consisting of many simple, densely interconnected, parallelly interacting processing elements (neurons or nodes) that are tied by adaptive weight connections (synaptic weights) and arranged in layers. The intelligence of ANNs lies in their ability to learn from patterns, process and store information, and use the knowledge abstracted from experiences to make their own decisions based on the data they have been exposed to. In general, the ANNs were designed to study the behavior of real, nonlinear, complex systems, and they are particularly effective in solving problems where the correlations between the dependent and independent variables are well-known. However, their precise description by classical mathematical methods is too complicated, too simplified, or impossible [22].

In the process of learning from representative patterns (training process), the ANN maps any complex relationship between the independent (input) and dependent (output) variables of the problem. The knowledge or rules abstracted from this mapping are (similarly to the human brain) distributed in parallel in the network structure in the form of weighted connections (weights), then generalized and subsequently used to predict or simulate the outputs for input data that were not part of the dataset used in the learning step. It is the set of distributed weights that represents the ANN model of the given problem [23].

Generally, the architecture of the most often used ANNs consists of at least three layers—one input layer, at least one hidden layer and one output layer—with an input, hidden and output set of neurons (multi-layer architecture). Each neuron of each layer is connected by the weights to all of the neurons in the higher layer and, through the bias, directly to the external environment. However, there are no connections between the neurons on the same layer, as with between the neurons, which are not in the adjacent layers (fully connected architecture). The information coming from the input layer is mapped, in a forward direction, to the output layer through all the hidden layers located between the input and output layers (feed-forward architecture). The formulation of the given problem determines the number of neurons in the input as well as the output layer, while the number of hidden layers and the number of hidden neurons (ANN topology) determine the ANN‘s capacity to be tuned during the learning using various types of learning algorithms (most often an error back-propagation algorithm, in which the weights are updated backwards, from output to input) [24]. The various types of ANNs with different neuron transfer or activation functions, which compute the output of a neuron based on its inputs, their architecture, and many learning algorithms are described in a number of available publications, e.g., in [25,26,27] or in our previous works [28,29,30]. Therefore, a generalized regression neural network (GRNN), which we used to model the temperature-dependence of curing characteristics of RBs with different carbon black (CB) filler contents, is briefly introduced in the next paragraph.

### 1.4. Generalized Regression Neural Networks

GRNNs are memory-based, probabilistic feed-forward ANNs with a simple dynamic structure. Due to their excellent features, such as a strong nonlinear mapping capability, high fault tolerance and robustness, they are often employed in various fields, especially in solving the function approximation or function regression problems. In general, GRNNs can approximate any continuous function between input and output data based on a finite number of training samples. Moreover, they share a favorable property, namely that they do not require an iterative training procedure, and so the speed of their one-pass learning algorithm is very high compared with the conventional, currently most commonly used error back-propagation ANNs [31].

GRNNs were proposed as a special modification of radial basis neural networks, and they are based on nonlinear regression analysis—namely, on the estimation of an unknown probability density function of a continuous random independent vector variable ***x*** ϵ *R^P^* and the corresponding random dependent vector variable ***y*** from a sequence of sample data [32]. Using a Parzen–Rosenblatt density estimator with a Gaussian kernel of specified width [33], the joint probability density function *φ_i_*(*x*, *y*) between each pair of scalar components *x*, *y* of the vectors ***x*** and ***y*** can be estimated from an observed sample dataset (*x_i_*, *y_i_*) according to the formula

(1) $$\varphi_{i}\left(x,y\right)=\frac{1}{{\left(2\pi \right)}^{\frac{P+1}{2}}\sigma^{P+1}}\frac{1}{M}\overset{M}{\underset{i=1}{\sum }}\exp\left[-\frac{{\left(x-x_{i}\right)}^{T}\left(x-x_{i}\right)}{2\sigma^{2}}\right]\exp\left[-\frac{{\left(y-y_{i}\right)}^{2}}{2\sigma^{2}}\right],$$

where *x_i_* and *y_i_* are the *i*th sample data points, *M* is the number of samples, *P* is the dimension of the variable ***x***, *σ* is the standard deviation or width coefficient of the Gaussian function, and the superscript ^T^ denotes the transpose operation of the vector [34].

The essence of GRNNs is to reconstruct the underlying regression function ***y***(***x***) between input and target data from the training samples. The best reconstruction of ***y***(***x***) with minimum variance *σ*^2^, or the regression of *y* relative to *x*, which predicts the most probable value of *y*(*x*) is given by the expected conditional mean value of *y* corresponding to the specified value of *x*, and it can be computed as:

(2) $$y\left(x\right)=\frac{\int y_{i}\varphi_{i}\left(x,y\right)dy_{i}}{\int \varphi_{i}\left(x,y\right)dy_{i}}.$$

After solving the integrals in Equation (2) and using Equation (1), the final expression for the predicted *y*(*x*), which is also called the Nadaraya–Watson kernel regression estimator due to its original statistical derivation [35], for a single-bandwidth GRNN is given by:

(3) $$y\left(x\right)=\frac{\sum_{i=1}^{M}y_{i}\exp\left(-\frac{D_{i}^{2}}{2\sigma^{2}}\right)}{\sum_{i=1}^{M}\exp\left(-\frac{D_{i}^{2}}{2\sigma^{2}}\right)},$$

where

(4) $$D_{i}^{2}\left(x,x_{i}\right)=\overset{R}{\underset{k=1}{\sum }}\|x_{k}-x_{ik}\|{}^{2}=\overset{R}{\underset{k=1}{\sum }}{\left(x_{k}-x_{ik}\right)}^{T}\left(x_{k}-x_{ik}\right)$$

is the squared Euclidean distance between the current input value *x_k_* and the corresponding *i*th training sample *x_ik_*, which is used as a measure of how well each training sample *x_ik_* can represent the position of the prediction point *x_k_*, and *R* represents the number of elements *x* of input vector ***x*** [36]. It is obvious from Equation (3) and Equation (4) that the predicted value *y*(*x*) is the nonlinearly weighted average of all training target values *y_i_* for training input cases *x_i_*, where the weighting factor of each *y_i_* is the Gaussian radial basis function (RBF)

(5) $$\psi_{i}\left(x,x_{i}\right)=\exp\left(-\frac{D_{i}^{2}\left(x,x_{i}\right)}{2\sigma^{2}}\right)$$

with a positive spread constant or smoothness parameter *σ* [37].

The spread constant *σ*, which directly affects the success of the GRNN in the prediction and determines its generalization performance, is the only adjustable parameter and it can be tuned by the training process to an optimum when the correlation between the actual and predicted value is high and the error between them becomes very small. In general, when the value of *σ* is moderate, all the training samples are taken into account, and the samples close to the predicted point are added to the *y*(*x*) computation with more weight. A larger *σ* may result in better generalization ability because the predicted *y*(*x*) approximates the mean of all training samples *y_i_*. On the other hand, when *σ* tends to 0, only a few training samples play a role in evaluating *y*(*x*) that is very close to them, so the GRNN’s generalization ability is poor. Too small *σ* values thus cause overfitting, where the model is able to predict data that it was trained on very well, but is unable to generalize and accurately predict data it has not seen before. At the same time, too large *σ* values cause underfitting of the model, where it is not even able to predict the data it was trained on, let alone data it has not seen before, respectively [38].

The typical GRNN topology structure, based on the above theory, consists of four neuron layers, including the input, pattern, summation, and output layer. GRNNs are feed-forward ANNs, so the signals always propagate from the first neuron layer to the last one. The number of neurons in the input and output layers corresponds to the number of *P* independent and *Q* dependent variables of the GRNN, respectively. The role of the input layer is to transport the received input data to all of the neurons in the fully connected pattern layer, where the number of neurons is equal to the number of training samples. The pattern layer processes the data in such a manner as to memorize the relationship between the input and target training samples (memory-based ANN [39]). The radial basis activation function is centered on a training sample *x_i_* for each neuron *i*, and its output is a measure of the distance *D_i_* of *x* from the *x_i_* according to Equation (4). However, before entering the RBFs, the distances *D_i_* are scaled by multiplication of their values by a bias *b*, usually the same for each neuron in the pattern layer (single-bandwidth GRNN). After scaling of each *D_i_*, the activation function *ψ_i_* (Equation (5)) takes the form of [40]:

(6) $$\psi_{i}\left(x,x_{i}\right)=\exp\left[-{\left(D_{i}\left(x,x_{i}\right)\;\times \;b\right)}^{2}\right]$$

The neuron bias that plays the role of the variance *σ*^2^ of *ψ_i_* Gaussian allows the sensitivity of the pattern layer neurons to be adjusted, and it can be computed directly from the GRNN parameter *σ* according to the formula [41]:

(7) $$b=\frac{\sqrt{-\ln\left(0.5\right)}}{\sigma }=\frac{0.8326}{\sigma }.$$

Each pattern layer neuron is connected to the two types of neurons in the summation layer: the *S_N_*-summation neuron, which computes the sum of the weighted outputs of the pattern layer:

(8) $$S_{N}=\overset{M}{\underset{i=1}{\sum }}y_{i}\psi_{i},$$

and the *S_D_*-summation neuron, which simply computes the unweighted sum of the output of each pattern layer neuron:

(9) $$S_{D}=\overset{M}{\underset{i=1}{\sum }}\psi_{i}.$$

The connection weight between the *i*th neuron in the pattern layer and the *S_N_*-summation neuron is the target output *y_i_* corresponding to the *i*th input sample *x_i_*, while for the *S_D_*-summation neuron, the connection weight is unity.

The output layer just performs the division between the output of the *S_N_*-summation neuron and that of the output of the *S_D_*-summation neuron, yielding the predicted *y*(*x*) as:

(10) $$y\left(x\right)=\frac{S_{N}}{S_{D}}.$$

Unlike error-back-propagation ANNs, which must be trained iteratively for many rounds to determine the connection weights between the neurons in different layers, the architecture and weights of GRNNs are determined when the input to the network is given. During GRNN training, each data sample is catered for as the average of normal distribution, and the output can be expressed according to Equation (10). Therefore, the training of GRNN represents the optimization of its spread constant *σ*, which is the only free parameter of the network [42]. Various optimization methods are currently used to find the optimal *σ*, such as the trial-and-error method, cross-validation, hold-out, hill-climbing, conjugate gradient, and others, including so-called soft computing methods of artificial intelligence, such as genetic algorithms [43]. The present work used the first of the named methods for its sufficiently high efficiency in solving the given function approximation problem.

## 2. Materials and Methods

### 2.1. Materials

The composition of the RBs was the following: 100 phr of styrene-butadiene rubber (SBR) grade SBR 1500 (Synthos Kralupy a.s., Kralupy nad Vltavou, Czech Republic); 3 phr of zinc oxide (ZnO) vulcanization activator (SlovZink a.s., Košeca, Slovakia); 1 phr of stearic acid vulcanization activator (Setuza a.s., Ústí nad Labem, Czech Republic); 1.75 phr of vulcanizing agent sulfur (S) type Crystex OT33 (Eastman Chemical company, Kingsport, TN, USA); 1 phr of N-tert-Butyl-2-benzothiazole-sulphenamide (TBBS) vulcanization accelerator (Duslo a.s., Šaľa, Slovakia); and 0 phr and 30–75 phr (with a steady increase of 5 phr) of CB filler grade N550 (Makrochem Sp. z o.o, Lublin, Poland).

### 2.2. Samples Preparation

The RBs were prepared by means of a two-stage mixing method. In the first step of mixing, the SBR was masticated for 3 min at a temperature of 90 ± 1 °C and rotor speed of 50 ± 1 rpm in a laboratory mixer Brabender Plastograph EC Plus (Brabender GmBH & Co.KG, Duisburg, Germany) with a mixing chamber volume of 80 cm^3^. Then, the ZnO was incorporated and mixed for 45 s, and subsequently, the CB was added and mixed for 3 min. Subsequently, the stearic acid was added and mixed for 30 s. The additional homogenization of prepared masterbatches (MBs) was carried out using a laboratory two-roll mill LaboWalz W150 (Voght Labormaschinen GmBH, Berlin, Germany) with 200 mm diameter and 400 mm working distance between the rolls at a temperature of 70 ± 1 °C. The speed of a slow roll was 24 rpm, and the gear ratio was 1:1.4. After allowing the MBs to cool for 24 h at room temperature, in the second step of the mixing procedure, they were initially blended for 3 min, then the S and TBBS were added and each one mixed for 1.5 min in the laboratory mixer with a rotor speed of 50 ± 1 rpm at temperature 90 ± 1 °C. The blended RBs were homogenized in the two-roll mill in the same conditions as in the first step of the mixing procedure, and then left to rest for 24 h at room temperature before the upcoming rheological analysis.

### 2.3. Rheological Analysis

An oscillating-disk rheometer RPA 2000 (Alfa Technologies Ltd., Akron, OH, USA) was utilized to obtain the cure curves and to determine the curing characteristics, such as minimum *M*_L_ and maximum *M*_H_ elastic torque values, scorch time *t*_s01_ and optimal cure time *t*_c90_ values for each RB with different contents of CB, as well as for an unfilled RB sample, individually. The rheological measurements were performed at cure temperatures of 165–210 °C with a steady increase of 5 °C, at an oscillating frequency of 1.67 Hz, and an oscillating angle of 1°. Subsequently, the acquired experimental data were subjected to ANN analysis in order to create a reliable model for predicting the curing characteristics of RBs.

### 2.4. Artificial Neural Network Modelling

The implementation of the ANN model for predicting the curing characteristics of RBs with different contents of CB filler at various cure temperatures was performed in the MATLAB^®^ software package, Version 9.0.0.341360 R2016a 64-bit, equipped with a Neural Network Toolbox (Math Works, Natic, MA, USA), that provides a number of built-in tools for sufficiently powerful and user-friendly work with ANNs of a wide range of types and architectures. The GRNN was used to solve the given function approximation problem, in particular for its extremely high learning rate and rapid convergence to optimal regression levels, even in the case of a small amount of training data [31].

## 3. Results and Discussion

### 3.1. Experimental Results

**Figure 1:**
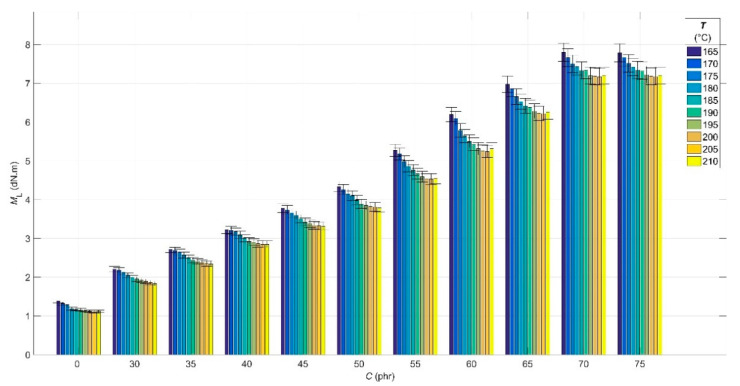
Minimum elastic torque values *M*_L_ for rubber blends with different carbon black filler *C* contents at various cure temperatures *T*.

**Figure 2:**
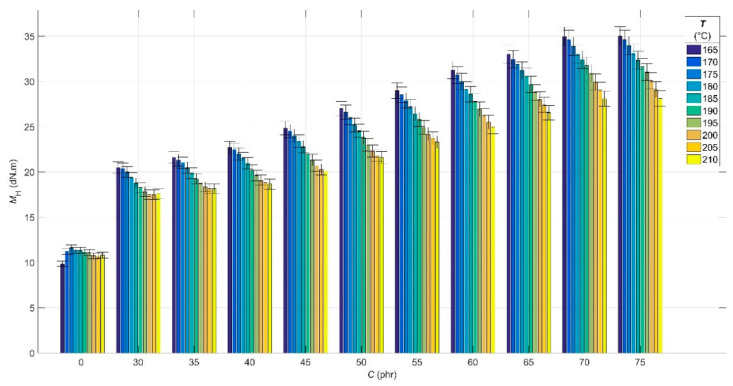
Maximum elastic torque values *M*_H_ for rubber blends with carbon black filler *C* contents at various cure temperatures *T*.

**Figure 3:**
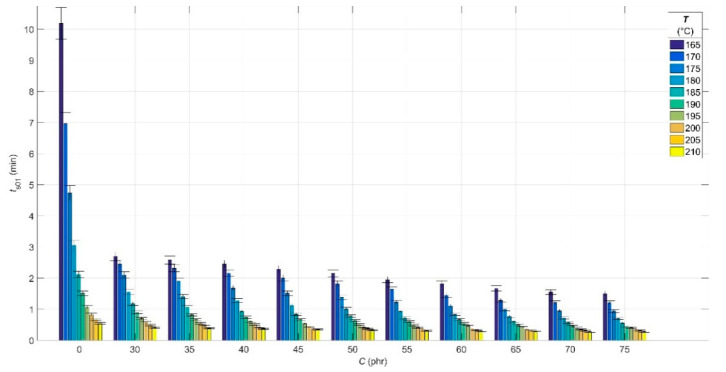
Scorch time values *t*_s01_ for rubber blends with different contents of carbon black filler *C* at various cure temperatures *T*.

**Figure 4:**
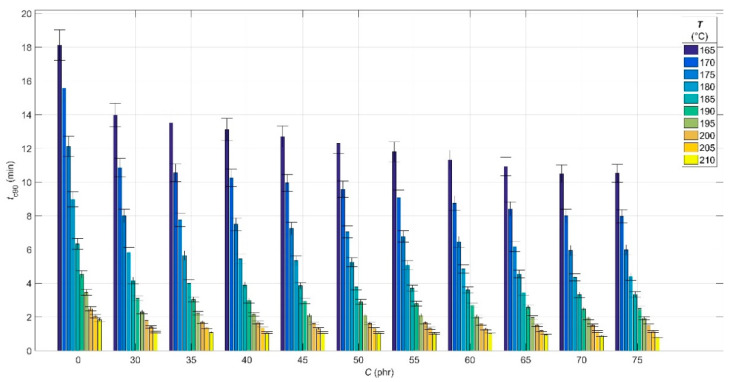
Optimal cure time values *t*_c90_ for rubber blends with different carbon black filler *C* contents at various cure temperatures *T*.

The results of the analysis of the experimental cure curves in the form of dependences of minimum *M*_L_ and maximum *M*_H_ elastic torque values, scorch time *t*_s01_ and optimal cure time *t*_c90_ values on CB contents *C* of 0 phr and in the range of 30–75 phr, and cure temperature *T* in the range of 165–210 °C are presented in Figure 1, Figure 2, Figure 3 and Figure 4, respectively.

From Figure 1, it can be seen that the *M*_L_, expressing the stiffness of the blend heated to a constant cure temperature at a constant pressure maintained in the measuring chamber of the rheometer, increases with the increase in the CB content in RB due to the limiting effect of the filler on the movement of the polymer chains of the rubber, which is the cause of the growth of its viscosity [44]. However, simultaneously with this effect of the CB filler, there is also an effect of a polymer matrix containing a pendant styrene group, which also increases its viscosity by restricting the movement of rubber chains [45]. At the same time, the increasing *M*_L_ or the increasing viscosity of the rubber with increasing CB filler contents causes the deterioration of the processability of the blend at higher CB filler contents in it [46].

The *M*_L_ value for CB contents up to 55 phr and cure temperatures up to 205 °C shows a decreasing trend, which is due to the increasing plasticization speed of the polymer matrix or decrease in its viscosity. At 210 °C and higher CB contents, a slight increase in *M*_L_ can be observed due to the flocculation of CB particles as a result of the so-called overheating of the blend and the oscillating motion of the rheometer disc, which allows their higher mobility, limiting the movement of the polymer chains of the rubber, leading to the observed increase in the *M*_L_ [47].

The dependence of *M*_H_ on CB contents (Figure 2) shows a similar trend as in the case of *M*_L_—with increasing CB filler contents in the blend, the *M*_H_ increases as a consequence of increasing its stiffness [48]. In contrast to *M*_L_, the *M*_H_ depends not only on the filler contents and the viscosity of the rubber matrix, but mainly on the number of cross-links formed in the vulcanization process. At the same time, the difference between the *M*_H_ and *M*_L_ determines the relative network density, which quantifies the number of cross-links formed [46]. In this case, the number of cross-links and relative network density of the blend can be attributed mainly to the effect of the vulcanization system because the cross-linking density was not specifically affected by the other raw materials needed to increase the cross-linking efficiency of CB filler. In the rubber industry, it is common to influence cross-linking efficiency and cross-linking density in the case of inorganic fillers such as silica using silanogran (Si69) or when using organic fillers and nanofillers based on polysaccharides as a cell of ionic liquids [49,50].

The presence of filler in the RB restricts the movement of the newly formed bonds, thus increasing its stiffness at the end of the vulcanization, which is proportional to the CB filler contents in it [51].

As the cure temperature increases, the *M*_H_ decreases due to the breakdown of polysulphide bonds, the first bonds formed in the sulphur vulcanization of rubber. Polysulphide bonds are very unstable thermally and convert to form more stable mono- as well as disulphide bonds. The disintegration of polysulfide bonds is also partially limited by the effect of the CB filler preventing the movement of the polymer chains of the rubber [52].

One effect of the presence of the CB filler in RB is the aforementioned overheating, which depends on both filler contents and cure temperature. At low concentrations (30–35 phr) and temperatures above 200 °C, the effect of overheating is less pronounced, leading to a lower rate of polysulphide bonds dissolution and manifested by a stabilization of *M*_H_ values [53].

The scorch time *t*_s01_ can be defined as the time beyond which the vulcanization begins and the involvement of the vulcanization accelerator in this process [54]. The effect of filler contents on the *t*_s01_ value is strongly visible in Figure 3, especially at lower filler contents up to 40 phr. This decrease in the 40–75 phr interval is less steep or slower. The CB particles make the blend more viscous, which causes it to heat up more when stressed in the rheometer, and hence also results in faster involvement of the accelerator in the chemical reactions and earlier onset of the beginning of the vulcanization process [53].

The values of *t*_s01_ decrease with increasing cure temperature due to the decrease in the viscosity of the rubber (Figure 1)—the higher mobility of its chains at higher temperatures thus contributes to the faster onset of the cross-linking reaction [54]. In the temperature interval of 200–210 °C and filler contents higher than 45 phr, a slighter decrease in *t*_s01_ values can be observed due to the overheating of the blend [55].

From Figure 4, it can be seen that the optimum cure time *t*_c90_—which is defined as the time required to reach 90% of the maximum torque value of *M*_H_ [56]—decreases with increasing CB filler contents in the blend, but this decrease is milder than in the case of *t*_s01_ (Figure 3). The effect of CB filler contents on *t*_c90_, increasing blend overheating and hence crosslink formation, is similar to that of *t*_s01_—higher blend overheating can achieve faster network formation in less time [56].

With increasing cure temperature, the decrease in *t*_c90_ is more pronounced, especially up to 200 °C. In the temperature interval 165–200 °C, there is a synergistic effect of temperature and filler contents, similar to *t*_s01_. As the cross-linking density of the rubber increases, the amount of the accelerator as well as the activator of vulcanization rapidly decreases, with the result being that at temperatures above 200 °C, the rate of vulcanization reactions is constant due to the lack of these two ingredients and the decrease in *t*_c90_ shows a linear trend [57].

Since the aim of this study is the intelligent modelling of the above-analyzed dependences of all four curing characteristics of RBs, we will deal with a detailed description of the creation of their GRNN model in the following sections, based on the theoretical considerations given in the introduction.

### 3.2. Pre-Processing of Experimental Data for ANN Analysis

The computer implementation of the ANN model requires a special pre-processing of the raw experimental data, which is described in detail below.

Six variables, which determine the results of the curing process of RBs, were considered for ANN analysis: the CB contents ***C*** = (*C*_1_*, C*_2_*, … C_m_*) in the RB and cure temperature ***T*** = (*T*_1_*, T*_2_*, … T_n_*) were used as ANN input data, while corresponding values of minimum ***M*_L_**(***C****, **T***) and maximum ***M*_H_**(***C****, **T***) elastic torque, scorch time ***t*_s01_**(***C****, **T***) and optimal cure time ***t*_c90_**(***C****, **T***) values were used as ANN target data.

The input and target data were stored as inputs and target parameters of the ANN in the form of ***Inputs*** = [***C***; ***T***] and ***Targets*** = [***M*_L_**; ***M*_H_**; ***t*_s01_**; ***t*_c90_**] data matrices, respectively, and they can be presented as:

(11) $$Inputs=\left(\begin{array}{ccccccccccccc} C_{1} & C_{1} & \ldots & C_{1} & C_{2} & C_{2} & \ldots & C_{2} & \ldots & C_{m} & C_{m} & \ldots & C_{m}\\ T_{1} & T_{2} & \ldots & T_{n} & T_{1} & T_{2} & \ldots & T_{n} & \ldots & T_{1} & T_{2} & \ldots & T_{n} \end{array}\right)$$

and

(12) $$Targets\;=\;\left(\begin{array}{ccccccccccccc} M_{H_{1}}^{1} & M_{H_{2}}^{1} & \ldots & M_{H_{n}}^{1} & M_{H_{1}}^{2} & M_{H_{2}}^{2} & \ldots & M_{H_{n}}^{2} & \ldots & M_{H_{1}}^{m} & M_{H_{2}}^{m} & \ldots & M_{H_{n}}^{m}\\ M_{L_{1}}^{1} & M_{L_{2}}^{1} & \ldots & M_{L_{n}}^{1} & M_{L_{1}}^{2} & M_{L_{2}}^{2} & \ldots & M_{L_{n}}^{2} & \ldots & M_{L_{1}}^{m} & M_{L_{2}}^{m} & \ldots & M_{L_{n}}^{m}\\ t_{s01_{1}}^{1} & t_{s01_{2}}^{1} & \ldots & t_{s01_{n}}^{1} & t_{s01_{1}}^{2} & t_{s01_{2}}^{2} & \ldots & t_{s01_{n}}^{2} & \ldots & t_{s01_{1}}^{m} & t_{s01_{2}}^{m} & \ldots & t_{s01_{n}}^{m}\\ t_{c90_{1}}^{1} & t_{c90_{2}}^{1} & \ldots & t_{c90_{n}}^{1} & t_{c90_{1}}^{2} & t_{c90_{2}}^{2} & \ldots & t_{c90_{2}}^{2} & \ldots & t_{c90_{1}}^{m} & t_{c90_{2}}^{m} & \ldots & t_{c90_{2}}^{m} \end{array}\right).$$

Because the inputs and targets of ANN are different physical quantities with different units and with a different range of values, for a more efficient optimization during the training, all the input and target data were normalized into the [0, 1] interval according to the formula [27]

(13) $$x_{\mathrm{norm}}=\frac{x-x_{\min}}{x_{\max}-x_{\min}},$$

where *x*, *x*_min_ and *x*_max_ represent the original data, their minimal and maximal values before normalization, respectively, and *x*_norm_ are the normalized data. The reverse of normalized ANN outputs to original data after simulation was performed according to the relationship [29]:

(14) $$x=x_{\min}+x_{\mathrm{norm}}\left(x_{\max}-x_{\min}\right).$$

The normalized data were then divided into a set of training data to create the ANN model and a test set of data to evaluate the predictive performance and generalization capability of the model [30]. The data for all the cure temperatures, but only the data corresponding to CB contents in the range from 30 phr to 70 phr with a steady increase of 10 phr and an unfilled RB sample, were used for training the model. Such an unusual division of data made it possible to reduce the number of training samples and thus also the number of measurements required while maintaining the high predictive performance of the model, which significantly increases its potential benefits for the rubber industry. In addition, a lower number of training samples reduces the likelihood of model overfitting [23]. However, the parallel reduction in the number of curing temperatures reduces the scope of the training data in such a way that leads to underfitting [24], so the model cannot provide any sufficiently reliable prediction.

The remaining data, corresponding to CB contents ranging from 35 to 75 phr with a steady increase of 10 phr, and cure temperatures in the range of 165–210 °C with a steady increase of 0.1 °C, which were not included in the training dataset, were used to evaluate the predictive performance and generalization capability of the model.

### 3.3. GRNN Training Algorithm

**Figure 5:**
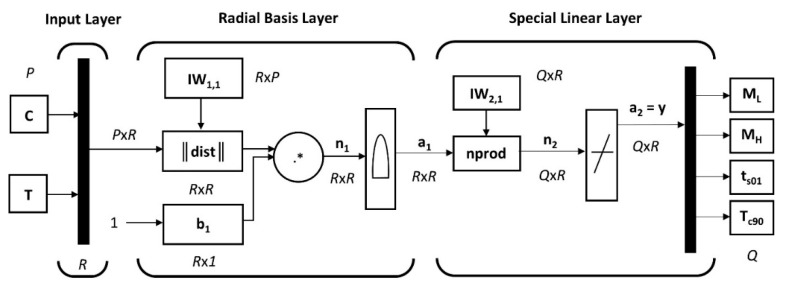
The scheme of the topological structure of GRNN model for prediction of the curing characteristics of rubber blends.

The topological structure of the developed GRNN model based on the MATLAB^®^ Neural Network Toolbox consists only of three neuron layers, namely, the input layer, a hidden RBF layer and a special linear output layer [58], and its scheme is shown in Figure 5.

The training process of the GRNN is as follows: once the *P* neurons of the input layer receive the input signal from the individual vectors ***C*** and ***T*** of the input training data matrix ***Inputs*** of size *P* × *R* (Equation (11)), the MATLAB^®^ built-in Euclidean distance weight function “*dist*” is used to compute the *R* × *R* Euclidean distance matrix ||***dist***|| (Equation (4)) between each pair of corresponding elements of *R* × *P* input weight matrix ***IW*_1,1_** and input training data matrix ***Inputs*** according to the relationship

(15) $$\|dist\|=\|IW_{1,1}-Inputs\|=dist\left(IW_{1,1},Inputs\right),$$

where the weight matrix ***IW*_1,1_** of RBF layer with *R* hidden neurons is set to a transposed input training data matrix ***Inputs***^T^. Then, the ||***dist***|| matrix is multiplied, element-by-element (symbol ‘.*’), by the *R* × 1 bias constant vector ***b*_1_** (Equation (7)) using the net input function “*netprod*” to provide the *R* × *R* net input

(16) $$n_{1}=\|dist\|\;.*b_{1}=netprod\left(\|dist\|,b_{1}\right)$$

to the “*radbas*” RBF transfer function (Equation (5)) that gives the *R* × *R* output of the RBF layer

(17) $$a_{1}=\exp\left(-n_{1}^{2}\right)=radbas\left(n_{1}\right).$$

A normalized dot product weight function “*normprod*” of the special linear output layer with *Q* neurons returns the *Q* × *R* dot product (symbol ‘.’)

(18) $$n_{2}=\frac{LW_{2,1}.a_{1}}{sum\left(a_{1}\right)}=normprod\left(LW_{2,1},a_{1}\right)$$

of the *Q* × *R* input weight matrix ***LW*_2,1_** and the input matrix ***a*_1_** normalized by the sum of all the elements of ***a*_1_** (Equation (10)), where the weight matrix of the output layer ***LW*_2,1_** is set as the *Q* × *R* target training data matrix ***Targets*** (Equation (12)).

A pure linear transfer function “*purelin*” takes the net input ***n*_2_** to produce the *Q* × *R* output matrix of the output layer

(19) $$a_{2}=n_{2}=purelin\left(n_{2}\right)$$

or

(20) $$a_{2}=purelin\left(normprod\left(LW_{2,1},radbas\left(neprod\left(dist\left(IW_{1,1},Inputs\right),b_{1}\right)\right)\right)\right),$$

where

(21) $$IW_{1,1}=Inputs^{T}=IW_{1,1}\left\{1,1\right\},LW_{2,1}=Targets=LW_{2,1}\left\{2,1\right\},$$

and ***b*_1_** = *b*{1} is given by Equation (7).

Finally, the output of the developed MATLAB^®^-based *P*-*R*-*Q-Q* (number of input-hidden-linear-output neurons) GRNN model, created and trained by the “*newgrnn*” built-in function, can be obtained by simulating it with inputs using the “*sim*” function—the training inputs are reapplied to the input layer of the trained network, and the desired outputs are compared with training targets at the output layer [58].

During the training process on the training dataset, an adjustable GRNN spread constant *σ* is set by a trial-and-error method to achieve the minimum prediction error of the trained model simulated with the test dataset.

**Figure 6:**
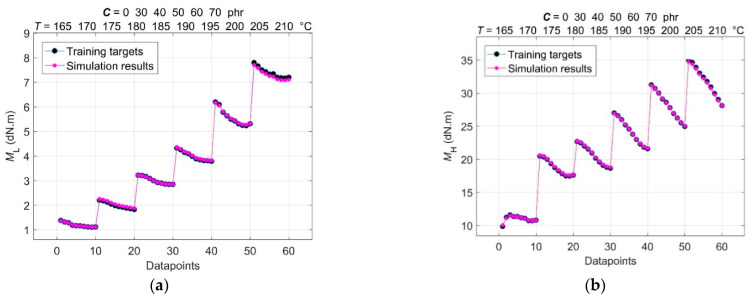
Comparison between training targets and network outputs for (**a**) minimum torque *M*_L_; (**b**) maximum torque *M*_H_.

**Figure 7:**
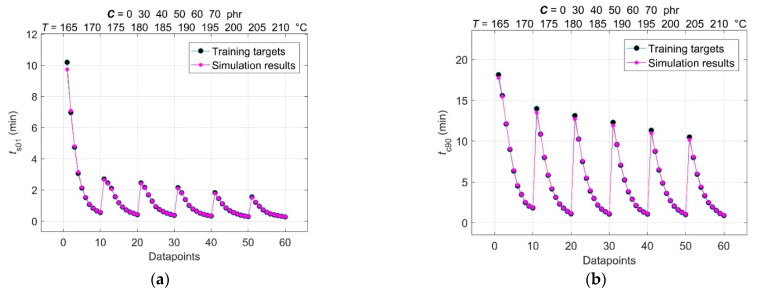
Comparison between training targets and network outputs for (**a**) scorch time *t*_s01_; (**b**) optimal cure time *t*_c90_.

The results of the comparison between training targets and network outputs for the optimal spread constant *σ* = 0.069, found by the trial-and-error method, and for the 2-60-4-4 GRNN model structure with a total number of (*P* + *Q*) × *R* or (2 + 4) × 60 training data points are shown in Figure 6 and Figure 7, respectively. All these curves show a high level of convergence without any underfitting [24].

### 3.4. Evaluation of the Goodness of the GRNN Model

**Figure 8:**
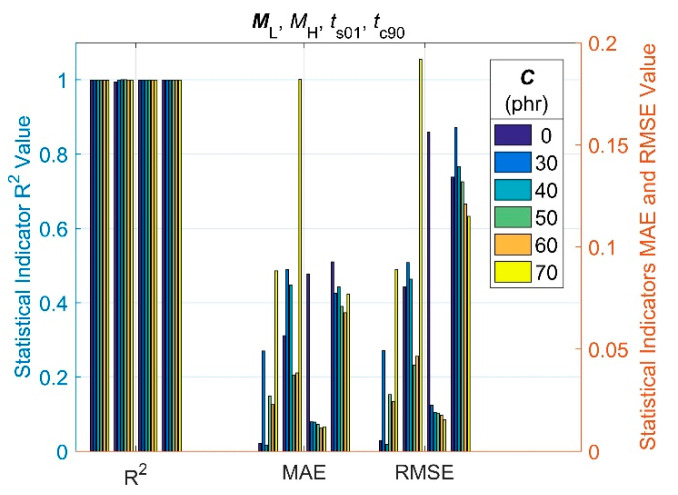
Statistical goodness parameters of the GRNN model.

The coefficient of determination (R^2^) between the modelled outputs and measurements of the training dataset, mean absolute error (MAE) and root mean square error (RMSE), as the most common statistical indicators, were used to provide a quantitative description of the goodness of the trained GRNN model estimates, and they are shown in Figure 8.

As can be seen from Figure 8, the RMSE and MAE parameters (which indicate residual errors between observed and predicted values) are very small, while R^2^ (representing the proportion of variability in the predicted results) is very close to its maximum value of 1 for all four curing characteristics. This means that the GRNN model is well trained, and its satisfactory predictive performance and generalization capability without any overfitting can be expected [59].

### 3.5. Evaluation of the Predictive Performance and Generalization Capability of the GRNN Model

**Figure 9:**
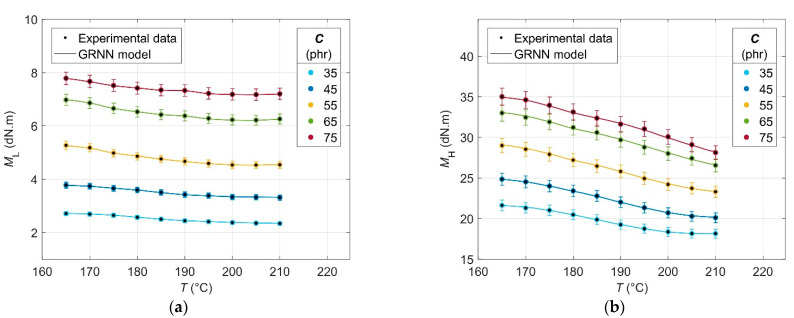
Comparison between the experimental and GRNN modelled data (**a**) minimum torque *M*_L_; (**b**) maximum torque *M*_H_.

**Figure 10:**
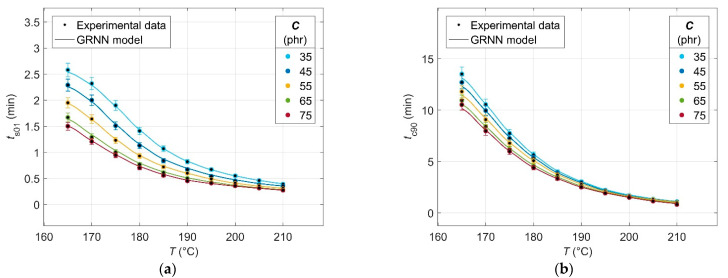
Comparison between the experimental and GRNN modelled data for (**a**) scorch time *t*_s01_; (**b**) optimal cure time *t*_c90_.

Evaluation of the predictive performance and generalization capability of the GRNN model was performed on a test dataset corresponding to CB contents in the range of 35–75 phr with a steady increase of 10 phr and cure temperatures in the range of 165–210 °C with a steady increase of 0.1 °C, which were not included in the training data. The comparison of experimental and modelled data is shown in Figure 9 and Figure 10.

Because the number of experimental and modelled data is different, the use of the abovementioned standard statistical indicators to quantify the generalization capability of the trained model is not possible. Therefore, the goodness of the model was expressed via the errors of the experimental data, which are shown in Figure 9 and Figure 10 by the error bars for each individual curing characteristic. The error for modelled *M*_L_ and *M*_H_ was found to be less than 3%, and the error of the modelled data for *t*_s01_ and *t*_c90_ does not exceed 5% of their experimental values, confirming an excellent predictive performance and generalization capability of the developed GRNN model.

## 4. Conclusions

In the presented work, a new artificial neural network-based model for predicting the curing characteristics of rubber blends with different contents of carbon black filler cured at various temperatures has been developed for the first time.

The variations of the minimum and maximum elastic torque, scorch time and optimal cure time with black carbon contents in the rubber blend and cure temperature have been analyzed in detail in the temperature range of 165–210 °C, with a steady increase of 5 °C, and in the carbon black filler contents in the range of 30–75 phr, with a steady increase of 5 phr, as well as for an unfilled rubber blend sample.

The carbon black contents and cure temperature have been used as input parameters, while all of the four above-mentioned curing characteristics have been considered to be the output parameters of the developed 2-60-4-4 generalized regression neural network model with an optimal spread constant of 0.069.

Less than 55% of experimental data have been used to significantly reduce the total number of input and target data points needed for training the model and avoid the overfitting and underfitting problems. The remaining data have been used as the test data for the evaluation of the predictive performance and generalization capability of the trained model.

A satisfactory agreement between the experimental and modelled values has been found for all four curing characteristics. The maximum error in the prediction for minimum and maximum elastic torque is less than 3%, and for scorch time and optimal cure time not exceeding 5% of their experimental values.

It can be concluded that the generalized regression neural network is a very powerful tool for intelligent modelling the curing process of rubber blends, even in the case of a small training dataset, and it can find widespread practical applications in the area of the rubber industry.

## Data Availability

Datasets related to this article can be found at https://data.mendeley.com/datasets/rfxtdz5pr2/2, an open-source online data repository hosted at Mendeley Data (Kopal et al. 2022).

## References

[1] Vayyaprontavida Kaliyathan A., Varghese K.M., Nair A.S., Thomas S. (2020). Rubber–rubber blends: A critical review. Prog. Rubber Plast. Recycl. Technol..

[2] Nakanishi Y., Mita K., Yamamoto K., Ichino K., Takenaka M. (2021). Effects of mixing process on spatial distribution and coexistence of sulfur and zinc in vulcanized EPDM rubber. Polymer.

[3] Kruželák J., Kvasničáková A., Hudec I. (2020). Peroxide curing systems applied for cross-linking of rubber compounds based on SBR. Adv. Ind. Eng. Polym. Res..

[4] Hansupalak N., Srisuk S., Wiroonpochit P., Chisti Y. (2016). Sulfur-Free Prevulcanization of Natural Rubber Latex by Ultraviolet Irradiation. Ind. Eng. Chem. Res..

[5] Haque M.E., Makuuchi K., Mitomo H., Yoshii F., Ikeda K. (2005). New Trend in Radiation Vulcanization of Natural Rubber Latex with a Low Energy Electron Beam. Polym. J..

[6] Kopal I., Vršková J., Bakošová A., Harničárová M., Labaj I., Ondrušová D., Valíček J., Krmela J. (2020). Modelling the Stiffness-Temperature Dependence of Resin-Rubber Blends Cured by High-Energy Electron Beam Radiation Using Global Search Genetic Algorithm. Polymers.

[7] Martin D., Ighigeanu D., Mateescu E., Craciun G., Ighigeanu A. (2002). Vulcanization of rubber mixtures by simultaneous electron beam and microwave irradiation. Radiat. Phys. Chem..

[8] Mutar M. (2010). A study in vulcanization of neoprene rubber (wrt) by polymethylol resin (resol). J. Al-Nahrain Univ. Sci..

[9] Karpeles R., Grossi A.G., Bhowmick A.K., Stephens H.L. (2001). EPDM Rubber Technology. Handbook of Elastomers.

[10] Hopmann C.H., Schmitz M. (2020). Data Acquisition and Process Monitoring as Enabler for Industry 4.0. Plastic Industry 4.0.

[11] Chang B.P.P., Gupta A., Muthuraj R., Mekonnen T. (2021). Bioresourced fillers for rubber composite sustainability: Current development and future opportunities. Green Chem..

[12] Yasin S., Hussain M., Zheng Q., Song Y. (2021). Large amplitude oscillatory rheology of silica and cellulose nanocrystals filled natural rubber compounds. J. Colloid Interface Sci..

[13] Labaj I., Skalková P., Dubec A., Janík R., Papučová I., Ondrušová D. Study of properties of elastomer blends based on natural rubber and chitosan. Proceedings of the 24th International Conference on Composite Structures.

[14] Dick J.S. (2009). Rubber Technology—Compounding and Testing for Performance.

[15] Abdelsalam A.A., Araby S., El-Sabbagh S.H., Abdelmoneim A., Hassan M.A. (2021). Effect of carbon black loading on mechanical and rheological properties of natural rubber/styrene-butadiene rubber/nitrile butadiene rubber blends. J. Thermoplast. Compos. Mater..

[16] Hossain M. (2010). Modelling and Computation of Polymer Curing. Ph.D. Thesis.

[17] Devi K.G., Rath M., Linh N.T.D. (2021). Artificial Intelligence Trends for Data Analytics Using Machine Learning and Deep Learning Approaches.

[18] ArulRaj K., Karthikeyan M., Narmatha D. (2021). A View of Artificial Neural Network Models in Different Application Areas. E3S Web Conf..

[19] Karaağaç B., İnal M., Deniz V. (2009). Artificial neural network approach for predicting optimum cure time of rubber compounds. Mater. Des..

[20] Karaağaç B., İnal M., Deniz V. (2012). Predicting optimum cure time of rubber compounds by means of ANFIS. Mater. Des..

[21] Lubura J.D., Kojić P., Pavličević J., Ikonić B., Omorjan R., Bera O. (2021). Prediction of rubber vulcanization using an artificial neural network. Hem. Ind..

[22] Du K.L., Swamy M.N.S. (2013). Neural Networks and Statistical Learning.

[23] Rao M.A., Srinivas J. (2003). Neural Networks: Algorithms and Applications.

[24] Paliwal M., Kumar U.A. (2009). Neural networks and statistical techniques: A review of applications. Expert Syst. Appl..

[25] Gambella C., Ghaddar B., Naoum-Sawaya J. (2021). Optimization problems for machine learning: A survey. Eur. J. Oper. Res..

[26] Hagan M.T., Demuth H.B., Beale M.H., De Jesús O. (2014). Neural Network Design.

[27] Liu X., Tian S., Tao F., Yu W. (2021). A review of artificial neural networks in the constitutive modeling of composite materials. Compos. B. Eng..

[28] Kopal I., Harničárová H., Valíček J., Kušnerová M. (2017). Modelling the temperature dependence of dynamic mechanical properties and visco-elastic behavior of thermoplastic polyurethane using artificial neural network. Polymers.

[29] Kopal I., Labaj I., Harničárová M., Valíček J., Hrubý D. (2018). Prediction of the tensile response of carbon black filled rubber blends by artificial neural network. Polymers.

[30] Kopal I., Harničárová M., Valíček J., Krmela J., Lukáč O. (2019). Radial basis function neural network-based modeling of the dynamic thermo-mechanical response and damping behavior of thermoplastic elastomer systems. Polymers.

[31] Dreyfus G. (2005). Neural Networks Methodology and Applications.

[32] Al-Mahasneh A.J., Anavatti S., Garratt M., Pratama M. (2018). Applications of General Regression Neural Networks in Dynamic Systems. Digital Systems, Asadpour, V., Ed..

[33] Farokhi F. (2020). Deconvoluting kernel density estimation and regression for locally differentially private data. Sci. Rep..

[34] Park J., Kun I. (2018). Fundamentals of Probability and Stochastic Processes with Applications to Communications.

[35] Pernot P., Savin A. (2020). Probabilistic performance estimators for computational chemistry methods: Systematic improvement probability and ranking probability matrix. I. Theory. J. Chem. Phys..

[36] O’Neill B. (2006). Elementary Differential Geometry.

[37] Wen H., Yan T., Liu Z., Chen D. (2021). Integrated neural network model with pre-RBF kernels. Sci. Prog..

[38] Specht D.F. (1991). A general regression neural network. IEEE Trans. Neural Netw..

[39] LeCun Y., Bengio Y., Hinton G. (2015). Deep learning. Nature.

[40] Sun X., Liu J., Zhu K., Hu J., Jiang X., Liu Y. (2019). Generalized regression neural network association with terahertz spectroscopy for quantitative analysis of benzoic acid additive in wheat flour. R. Soc. Open Sci..

[41] Sharkawy A.N. (2020). Principle of neural network and its main types. J. Adv. Appl. Comput. Math..

[42] Rajasekaran S., Pai G.V. (2004). Neural Networks, Fuzzy Logic and Genetic Algorithms.

[43] Huang Y.C., Liao H.S. (2020). Building prediction model for a machine tool with genetic algorithm optimization on a general regression neural network. J. Intell. Fuzzy Syst..

[44] Al-Nesrawy S.H., Al-Maamori M.H., Japor H. (2016). Effect of temperature on rheological properties of sbr compounds reinforced by some industrial scraps as a filler. Int. J. Chem. Sci..

[45] Arayapranee W., Rempel G.L. (2013). Effects of polarity on the filler-rubber interaction and properties of silica filled grafted natural rubber composites. J. Polym..

[46] Chalid M., Husnil Y.A., Puspitasari S., Cifriadi A. (2020). Experimental and Modelling Study of the Effect of Adding Starch-Modified Natural Rubber Hybrid to the Vulcanization of Sorghum Fibers-Filled Natural Rubber. Polymers.

[47] Sattayanurak S., Sahakaro K., Kaewsakul W., Dierkes W.K., Reuvekamp L.A., Blume A., Noordermeer J.W. (2020). Synergistic effect by high specific surface area carbon black as secondary filler in silica reinforced natural rubber tire tread compounds. Polym. Test..

[48] Ghosh J., Ghorai S., Jalan A.K., Roy M., De D. (2018). Manifestation of Accelerator Type and Vulcanization System on the Properties of Silica-reinforced SBR/devulcanize SBR blend Vulcanizates. Adv. Polym. Technol..

[49] Hussain M., Yasin S., Akram M.A., Xu H., Song Y., Zheng Q. (2019). Influence of Ionic Liquids on Structure and Rheological Behaviors of Silica-Filled Butadiene Rubber. Ind. Eng. Chem. Res..

[50] Yasin S., Hussain M., Zheng Q., Song Y. (2021). Effects of ionic liquid on cellulosic nanofiller filled natural rubber bionanocomposites. J. Colloid Interface Sci..

[51] Shanks R.A., Kong I., Visakh P., Thomas S., Chandra A., Mathew A. (2013). General Purpose Elastomers: Structure, Chemistry, Physics and Performance. Advances in Elastomers I. Advanced Structured Materials.

[52] Kurian T., George K.E., Francis D.J. (1988). Effect of vulcanization temperature on the cure characteristics and vulcanizate properties of natural rubber and styrene-butadiene rubber. Angew. Makromolek. Chem..

[53] Ramesan M.T. (2005). The effects of filler content on cure and mechanical properties of dichlorocarbene modified styrene butadiene rubber/carbon black composites. J. Polym. Res..

[54] Joseph A.M., George B., Madhusoodanan K.N., Alex R. (2017). Cure characteristics of devulcanized rubber: The issue of low scorch. Rubber Chem. Technol..

[55] Sadequl A.M., Ishiaku U.S., Ismail H., Poh B.T. (1998). The effect of accelerator/sulfur ratio on the scorch time of epoxidized natural rubber. Eur. Polym. J..

[56] Khimi S.R., Pickering K.L. (2014). A new method to predict optimum cure time of rubber compound using dynamic mechanical analysis. J. Appl. Polym. Sci..

[57] Maciejewska M., Siwek M. (2020). The Influence of Curing Systems on the Cure Characteristics and Physical Properties of Styrene–Butadiene Elastomer. Materials.

[58] Ploskas N., Samaras N. (2016). GPU Programming in MATLAB.

[59] de Bragança Pereira B., Rao C.R., de Oliveira F.B. (2020). Statistical Learning Using Neural Networks: A Guide for Statisticians and Data Scientists with Python.

